# Background Ionizing Radiation and the Risk of Childhood Cancer: A Census-Based Nationwide Cohort Study

**DOI:** 10.1289/ehp.1408548

**Published:** 2015-02-23

**Authors:** Ben D. Spycher, Judith E. Lupatsch, Marcel Zwahlen, Martin Röösli, Felix Niggli, Michael A. Grotzer, Johannes Rischewski, Matthias Egger, Claudia E. Kuehni, for the Swiss Pediatric Oncology Group, the Swiss National Cohort Study Group

**Affiliations:** 1 Institute of Social and Preventive Medicine (ISPM), University of Bern, Bern, Switzerland; 2 Swiss Tropical and Public Health Institute, Basel, Switzerland; 3 University of Basel, Basel, Switzerland; 4 Department of Oncology, University Children’s Hospital Zurich, Zurich, Switzerland; 5 Department of Oncology/Hematology, Children’s Hospital, Cantonal Hospital Lucerne, Lucerne, Switzerland

## Abstract

**Background:**

Exposure to medium or high doses of ionizing radiation is a known risk factor for cancer in children. The extent to which low-dose radiation from natural sources contributes to the risk of childhood cancer remains unclear.

**Objectives:**

In a nationwide census-based cohort study, we investigated whether the incidence of childhood cancer was associated with background radiation from terrestrial gamma and cosmic rays.

**Methods:**

Children < 16 years of age in the Swiss National Censuses in 1990 and 2000 were included. The follow-up period lasted until 2008, and incident cancer cases were identified from the Swiss Childhood Cancer Registry. A radiation model was used to predict dose rates from terrestrial and cosmic radiation at locations of residence. Cox regression models were used to assess associations between cancer risk and dose rates and cumulative dose since birth.

**Results:**

Among 2,093,660 children included at census, 1,782 incident cases of cancer were identified including 530 with leukemia, 328 with lymphoma, and 423 with a tumor of the central nervous system (CNS). Hazard ratios for each millisievert increase in cumulative dose of external radiation were 1.03 (95% CI: 1.01, 1.05) for any cancer, 1.04 (95% CI: 1.00, 1.08) for leukemia, 1.01 (95% CI: 0.96, 1.05) for lymphoma, and 1.04 (95% CI: 1.00, 1.08) for CNS tumors. Adjustment for a range of potential confounders had little effect on the results.

**Conclusions:**

Our study suggests that background radiation may contribute to the risk of cancer in children, including leukemia and CNS tumors.

**Citation:**

Spycher BD, Lupatsch JE, Zwahlen M, Röösli M, Niggli F, Grotzer MA, Rischewski J, Egger M, Kuehni CE, for the Swiss Pediatric Oncology Group and the Swiss National Cohort. 2015. Background ionizing radiation and the risk of childhood cancer: a census-based nationwide cohort study. Environ Health Perspect 123:622–628; http://dx.doi.org/10.1289/ehp.1408548

## Introduction

Ionizing radiation is a known risk factor for cancer [United Nations Scientific Committee on the Effects of Atomic Radiation (UNSCEAR) 2006]. For a given radiation dose, children are at a greater risk than adults (UNSCEAR 2013). Ionizing radiation is the only established environmental risk factor for childhood leukemia and tumors of the central nervous system (CNS), the two most common tumor types in childhood (Belson et al. 2007; Wiemels 2012; Wrensch et al. 2002). Evidence for the carcinogenic effects of ionizing radiation in children comes mainly from studies of exposure to moderate or high doses from atomic bombs or therapeutic radiation (Wakeford 2013; Wrensch et al. 2002). It remains unclear whether dose–response relationships observed in these study populations extend to lower doses from more widespread exposures such as diagnostic radiology or natural background radiation (Wakeford 2013).

Natural background radiation is ubiquitous and, for most people, the main source of radiation exposure (UNSCEAR 2000). About a third of this is attributable to cosmic rays and terrestrial gamma radiation whereas the rest is due to inhalation (mainly indoor radon) and ingestion of radionuclides (UNSCEAR 2000). Whereas the effective dose from radon is delivered primarily to the respiratory system, terrestrial gamma and cosmic rays dominate doses to the red bone marrow (Kendall et al. 2009), the primary site of leukemia initiation. In Switzerland, exposure levels of the resident population to background radiation vary considerably due to the relatively high radioactivity of crystalline rocks of the central Alpine massif compared with the sedimentary northern Alpine Foreland (Jura, Molasse Basin) (Rybach et al. 1996, 2002).

Most previous studies on the risk of childhood cancer and background ionizing radiation from terrestrial gamma or cosmic rays were ecological. Results from these studies were heterogeneous. Most of them showed little or no evidence of an association (Auvinen et al. 1994; Evrard et al. 2006; Mason and Miller 1974; Muirhead et al. 1992; Richardson et al. 1995; Tirmarche et al. 1988), and others suggested a positive (Hatch and Susser 1990; Knox et al. 1988) or even a negative association (Frigerio et al. 1973). Few studies to date have used individual data (Axelson et al. 2002; Kendall et al. 2013; UK Childhood Cancer Study Investigators 2002b). A case–control study from the United Kingdom using measurements made in children’s homes found no evidence of an association (UK Childhood Cancer Study Investigators 2002b). Recently, a record-based case–control study from the United Kingdom found evidence of an increasing risk with cumulative gamma-ray dose for childhood leukemia but not for other cancer types (Kendall et al. 2013).

Given the limited and conflicting evidence from previous studies, we used a nationwide cohort study to investigate the association between external ionizing radiation from cosmic and terrestrial sources and incidence of childhood cancer and its major diagnostic groups in Switzerland. Geocoded residential locations at census time points were available for the entire population, and a spatial model with separate components for terrestrial gamma radiation and the directly ionizing component of cosmic radiation was used for exposure assessment. Cases were identified from the Swiss Childhood Cancer Registry (SCCR; http://www.childhoodcancerregistry.ch/) (Michel et al. 2008).

## Methods

*Population*. Our study included the Swiss resident population < 16 years of age. Data collected on these children during national censuses in 1990 and 2000, including georeferenced residential locations and demographic and socioeconomic information, were obtained from the Swiss National Cohort (SNC; http://www.swissnationalcohort.ch/) (Bopp et al. 2009). The SNC is a research platform based on nationwide individual record linkage between different censuses and mortality and migration records. This linkage allows calculating follow-up time for all individuals registered in the two censuses (Bopp et al. 2009; Spoerri et al. 2010). Birth weight and birth order were obtained through record linkage with the national birth registry. We excluded children whose residential locations were unknown or uncertain or could not be georeferenced to within 100 m.

Cases of childhood cancer were identified from the SCCR. The SCCR has an estimated completeness of > 90% for cancers in children < 16 years old diagnosed in Switzerland since 1985 (Michel et al. 2008). We included all cases with a tumor classified according to the *International Classification of Childhood Cancer, Third Edition* (ICCC-3; http://seer.cancer.gov/iccc/) (Steliarova-Foucher et al. 2005). The SCCR collects residential address histories of patients from diagnosis back to birth, allowing us to obtain residence at census. Addresses were geocoded using a list of georeferenced building addresses from the Swiss postal system (GeoPost) or manually using the geoportal maintained by the Federal Office of Topography (swisstopo) at http://map.geo.admin.ch. We used probabilistic record linkage (G-LINK 2.3; Statistics Canada; http://www5.statcan.gc.ca/olc-cel/olc.action?lang=en&ObjId=10H0036&ObjType=22) to link cases with children from the SNC based on the variables sex, date of birth, maternal and paternal dates of birth, geocoded residence at census, municipality of residence at census and at birth, and nationality. This study is based on register data, and informed consent was not required. The SNC was approved by the ethics committees of the Cantons of Bern and Zurich and by the Federal Data Protection Office (http://www.edoeb.admin.ch/?lang=en).

*Outcomes*. We limited analyses to major diagnostic categories: any cancer (all ICCC-3 diagnostic groups), leukemias (ICCC-3 diagnostic group I), acute lymphoblastic leukemias (ALL) (I.a), lymphomas (II), and tumors of the CNS tumors (III), which include malignant and nonmalignant intracranial and intraspinal tumors. We also analyzed other malignant tumors comprising all remaining ICCC-3 diagnostic groups (IV–XII).

*Exposure assessment*. We estimated exposure to external background radiation as total dose rates at children’s homes from cosmic and terrestrial sources based on a previously developed exposure model (Rybach et al. 1996, 2002). This model estimates total dose rates for each cell of a 2 km × 2 km grid as the sum of three separately estimated components: the directly ionizing component of cosmic radiation, natural terrestrial gamma radiation, and artificial terrestrial radiation. The cosmic dose rate is calculated as a function of altitude. Grid values were obtained by averaging topographic altitude within grid cells using a digital terrain model. The natural terrestrial component combines airborne gamma-ray spectrometry (about 10% of the country’s surface surveyed by helicopter), *in situ* gamma-ray spectrometry (166 sites), *in situ* dose rate measurements using ionization chambers (837 sites), and laboratory measurements of rock and soil samples from 612 sites. These measurements span the time period from the early 1960s to mid-1990s. In addition to airborne measurements, a total of 1,615 ground data points were available; these correspond to about 1 point per 25 km^2^. The model did not account for temporal variations in natural radiation, for example, due to snow cover or sun activity. The artificial terrestrial component mainly reflects ^137^Cs (cesium) deposition originating from the Chernobyl accident and is based on airborne and *in situ* measurements taken after 1987. Grid cell dose rates for the terrestrial components were interpolated from the available data points using the inverse distance method and a search radius of 12 km. More details on measurements and calibration procedures are provided elsewhere (Rybach et al. 1996).

*Potential confounders*. We considered the following potential confounding factors: traffic-related air pollution (proxied by distance to nearest highway), electromagnetic fields from radio and TV transmitters (field strength based on a geographic model) (Hauri et al. 2014), and from high-voltage power lines (distance to nearest 380 kV or 220 kV power line), degree of urbanization of municipality (urban, peri-urban, rural), socioeconomic status based on the Swiss neighborhood index of socioeconomic position (Swiss-SEP) (Panczak et al. 2012), education of household reference person (compulsory, secondary, tertiary), and crowding (number of persons per room), birth weight, and birth order of the child.

*Statistical analyses*. We investigated incidence of childhood cancer by total dose rate using time to event analyses with age as the underlying time scale. Follow-up time began at the first census in which a child was recorded (entry time) and ended on the earliest of following events: diagnosis, death, emigration, the child’s 16th birthday, administrative censoring on 31 December 2008. Exposure was based on residential location at census. For a child appearing in both censuses but living at a different location in 2000 than in 1990, the 1990 exposure was updated in 1995 or 2000 depending on whether or not the child lived at the new location 5 years before census 2000 (information from census questionnaire). Total dose rate was categorized into regular intervals of 50 nSv/hr with subsequent regrouping such that no interval contained < 1% of the census populations. This resulted in the following categorization: < 100 nSv/hr, 100 to < 150, 150 to < 200, ≥ 200 nSv/hr. We estimated hazard ratios (HR) for different exposure categories using Cox proportional hazards models. All models were adjusted for sex and birth year, and in separate models we adjusted for the other potential confounders. We also ran trend analyses using a linear exposure term. To explore potential effects of misclassification due to residential mobility, we restricted time-to-event analyses to children who had a stable place of residence up to entry into the cohort, defined as those reporting at entry to have had the same residence 5 years earlier or, if these data were missing (e.g., for children < 5 years of age at census), reporting to have lived in the same municipality at birth.

We repeated the trend analyses using cumulative dose instead of dose rate. Cumulative dose was calculated by integrating dose rate over time since birth. To conduct these analyses, we created a nested case–control data set by randomly sampling 100 controls per case from among those at risk at the time of the case’s failure. We then calculated cumulative doses and fitted conditional logistic regression models conditioning on case–controls sets. This procedure is asymptotically equivalent to Cox proportional hazards regression using the full cohort (Goldstein and Langholz 1992). All analyses were done using Stata version 12.1 (StataCorp, College Station, TX, USA).

## Results

**Figure 1:**
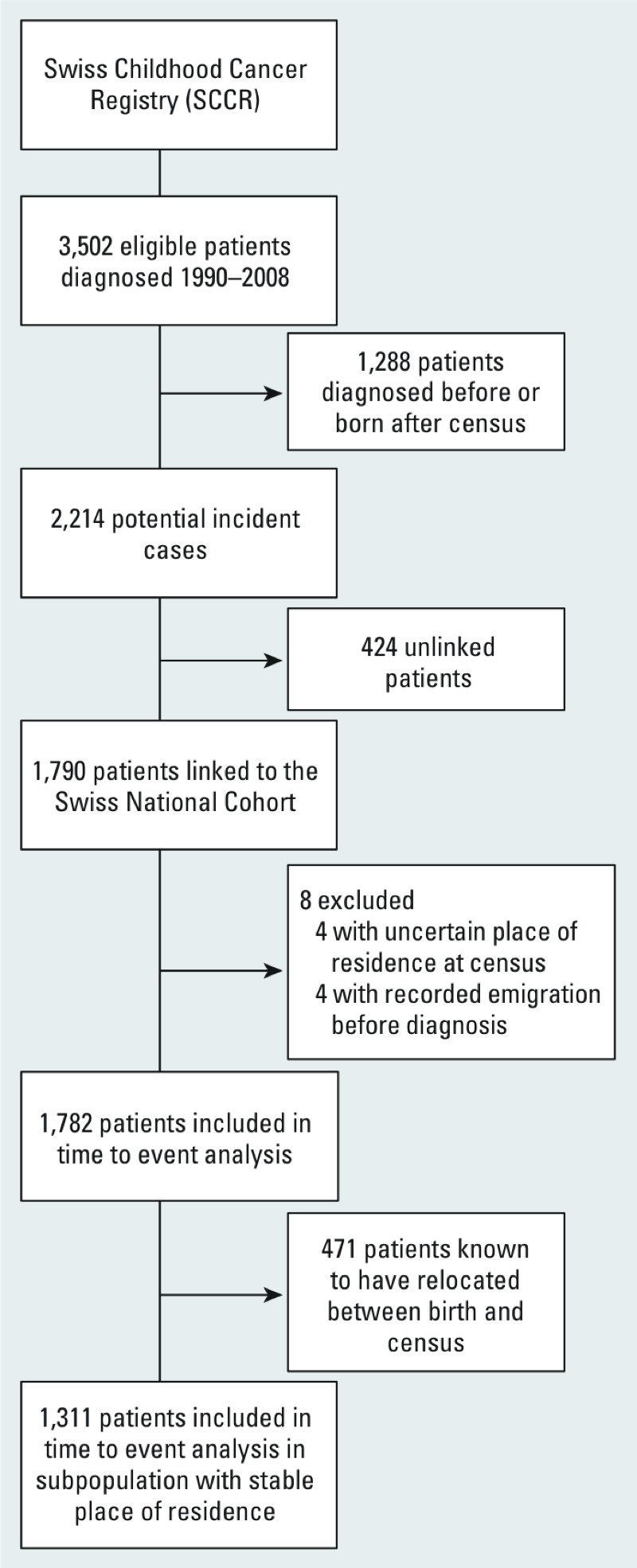
Flow chart of childhood cancer cases included in analyses.

*Characteristics of the study population*. Of 3,502 eligible patients in the SCCR who were diagnosed between the census in 1990 and the end of 2008, 1,782 could be included in time-to-event analyses, and of these 1,311 belonged to the subcohort of children with stable place of residence (Figure 1). The distribution of diagnoses among eligible and included cases is shown in Supplemental Material, Table S1. Because included cases were required to be without diagnosis at census, they tended to be older at diagnosis and hence included slightly fewer leukemia and more lymphoma and CNS cases compared with all eligible cases.

The SNC included 2,129,264 children < 16 years of age at census. Of these, 34,371 were excluded due to uncertain residence and 1,233 did not contribute person time (mostly because they were linked to an SCCR case diagnosed before census) leaving 2,093,660 (98.3%) for time-to-event analyses. These children had a mean age of 7.0 years at entry into the cohort, that is, the first census they were registered in. They were followed-up for a mean of 7.7 years and accrued 16.1 million person-years at risk. Follow-up time ended due to emigration or death in 47,119 children (2.6% of those included). At child’s entry into the cohort, the mean dose rate of external background radiation was 109 nSv/hr (median, 103 nSv/hr; range, 55–383 nSv/hr; interquartile range, 95–112 nSv/hr). On average, natural terrestrial radiation contributed 54 nSv/hr, cosmic radiation 45 nSv/hr, and artificial terrestrial radiation 8 nSV/hr. In terms of exposure variability, natural terrestrial radiation is most relevant (see Supplemental Material, Figure S1). Table 1 reports other characteristics of the study population at entry into the cohort according to categories of total external background radiation. Compared with the least exposed group, highly exposed children tended to live in more rural areas and in neighborhoods of lower SEP. For instance, 53% of children exposed to a dose rate of ≥ 200 nSv/hr belonged to the lowest SEP quintile compared with 26% of those exposed to < 100 nSv/hr. Children with higher exposure also tended to be more exposed to highways and electromagnetic fields from high voltage power lines and broadcast transmitters.

**Table 1:** Characteristics of study population by exposure to external background radiation.

Characteristic	Exposure category [*n* (%)]				*p-*Value^*a*^
	< 100 nSv/hr*n* = 806,450 (100.0%)	100 to < 150 nSv/hr*n* = 1,146,470 (100.0%)	150 to < 200 nSv/hr*n* = 119,245 (100.0%)	≥ 200 nSv/hr*n* = 21,495 (100.0%)	
Sex
Male	413,816 (51.3)	587,551 (51.2)	61,002 (51.2)	10,980 (51.1)	0.620
Female	392,634 (48.7)	558,919 (48.8)	58,243 (48.8)	10,515 (48.9)
Year of birth
1974–1979	143,378 (17.8)	206,716 (18.0)	22,720 (19.1)	3,884 (18.1)	< 0.001
1980–1984	147,055 (18.2)	210,097 (18.3)	21,658 (18.2)	4,072 (18.9)
1985–1989	171,858 (21.3)	247,084 (21.6)	24,959 (20.9)	4,636 (21.6)
1990–1994	167,075 (20.7)	234,456 (20.5)	24,091 (20.2)	4,398 (20.5)
1995–2000	177,084 (22.0)	248,117 (21.6)	25,817 (21.7)	4,505 (21.0)
Degree of urbanization
Urban	142,366 (17.7)	335,881 (29.3)	22,915 (19.2)	913 (4.2)	< 0.001
Peri-urban	366,564 (45.5)	526,711 (45.9)	52,251 (43.8)	7,214 (33.6)
Rural	297,520 (36.9)	283,878 (24.8)	44,079 (37.0)	13,368 (62.2)
Swiss-SEP index
1st quintile (low SEP)	210,686 (26.1)	297,632 (26.0)	56,522 (47.4)	11,423 (53.1)	< 0.001
2nd quintile	172,663 (21.4)	227,926 (19.9)	30,711 (25.8)	5,006 (23.3)
3rd quintile	161,461 (20.0)	214,197 (18.7)	18,456 (15.5)	3,098 (14.4)
4th quintile	146,832 (18.2)	209,000 (18.2)	10,398 (8.7)	1,568 (7.3)
5th quintile (high SEP)	112,837 (14.0)	194,929 (17.0)	2,687 (2.3)	351 (1.6)
Missing	1,971 (0.2)	2,786 (0.2)	471 (0.4)	49 (0.2)
Education level of head of household
Compulsory or less	138,987 (17.2)	210,295 (18.3)	24,486 (20.5)	3,502 (16.3)	< 0.001
Secondary level	407,213 (50.5)	555,474 (48.5)	64,151 (53.8)	13,056 (60.7)
Tertiary level	235,179 (29.2)	341,226 (29.8)	27,434 (23.0)	4,390 (20.4)
Not known	25,071 (3.1)	39,475 (3.4)	3,174 (2.7)	547 (2.5)
Persons per room (tertiles)
< 0.82	297,857 (36.9)	405,454 (35.4)	39,882 (33.4)	7,372 (34.3)	< 0.001
0.82 to < 1.08	296,570 (36.8)	417,782 (36.4)	44,575 (37.4)	7,929 (36.9)
≥ 1.08	212,023 (26.3)	323,234 (28.2)	34,788 (29.2)	6,194 (28.8)
Birth weight [g (tertiles)]
< 3,152	152,649 (18.9)	216,703 (18.9)	24,864 (20.9)	4,945 (23.0)	< 0.001
3,152 to < 3,541	152,019 (18.9)	209,712 (18.3)	22,582 (18.9)	4,406 (20.5)
≥ 3,541	156,445 (19.4)	212,081 (18.5)	20,558 (17.2)	3,844 (17.9)
Missing	345,337 (42.8)	507,974 (44.3)	51,241 (43.0)	8,300 (38.6)
Birth sequence
1st	193,045 (23.9)	272,626 (23.8)	29,287 (24.6)	5,581 (26.0)	< 0.001
2nd	168,655 (20.9)	233,509 (20.4)	25,130 (21.1)	4,825 (22.4)
3rd or later	85,261 (10.6)	109,151 (9.5)	11,207 (9.4)	2,343 (10.9)
Missing	359,489 (44.6)	531,184 (46.3)	53,621 (45.0)	8,746 (40.7)
Distance to nearest highway (m)
< 100	10,756 (1.3)	16,325 (1.4)	1,317 (1.1)	397 (1.8)	< 0.001
100 to < 250	28,945 (3.6)	47,372 (4.1)	5,318 (4.5)	1,072 (5.0)
250 to < 500	62,115 (7.7)	97,023 (8.5)	11,792 (9.9)	1,578 (7.3)
≥ 500	704,634 (87.4)	985,750 (86.0)	100,818 (84.5)	18,448 (85.8)
Distance to high-voltage power line (m)
< 100	6,066 (0.8)	7,198 (0.6)	2,203 (1.8)	588 (2.7)	< 0.001
100 to < 250	17,212 (2.1)	19,262 (1.7)	5,536 (4.6)	2,183 (10.2)
250 to < 500	41,979 (5.2)	52,866 (4.6)	11,736 (9.8)	3,281 (15.3)
≥ 500	741,193 (91.9)	1,067,144 (93.1)	99,770 (83.7)	15,443 (71.8)
EMF from broadcast transmitters (V/m)^*b*^
< 0.05	719,804 (89.3)	953,221 (83.1)	87,381 (73.3)	18,990 (88.3)	< 0.001
0.05 to < 0.2	67,901 (8.4)	142,217 (12.4)	19,705 (16.5)	1,019 (4.7)
≥ 0.2	17,390 (2.2)	48,744 (4.3)	11,900 (10.0)	1,454 (6.8)
Missing	1,355 (0.2)	2,288 (0.2)	259 (0.2)	32 (0.1)
Abbreviations: SEP, socioeconomic position; EMF, electromagnetic fields.^***a***^From chi-square tests. ^***b***^Modeled field strength from radio and TV transmitters.					

**Figure 2:**
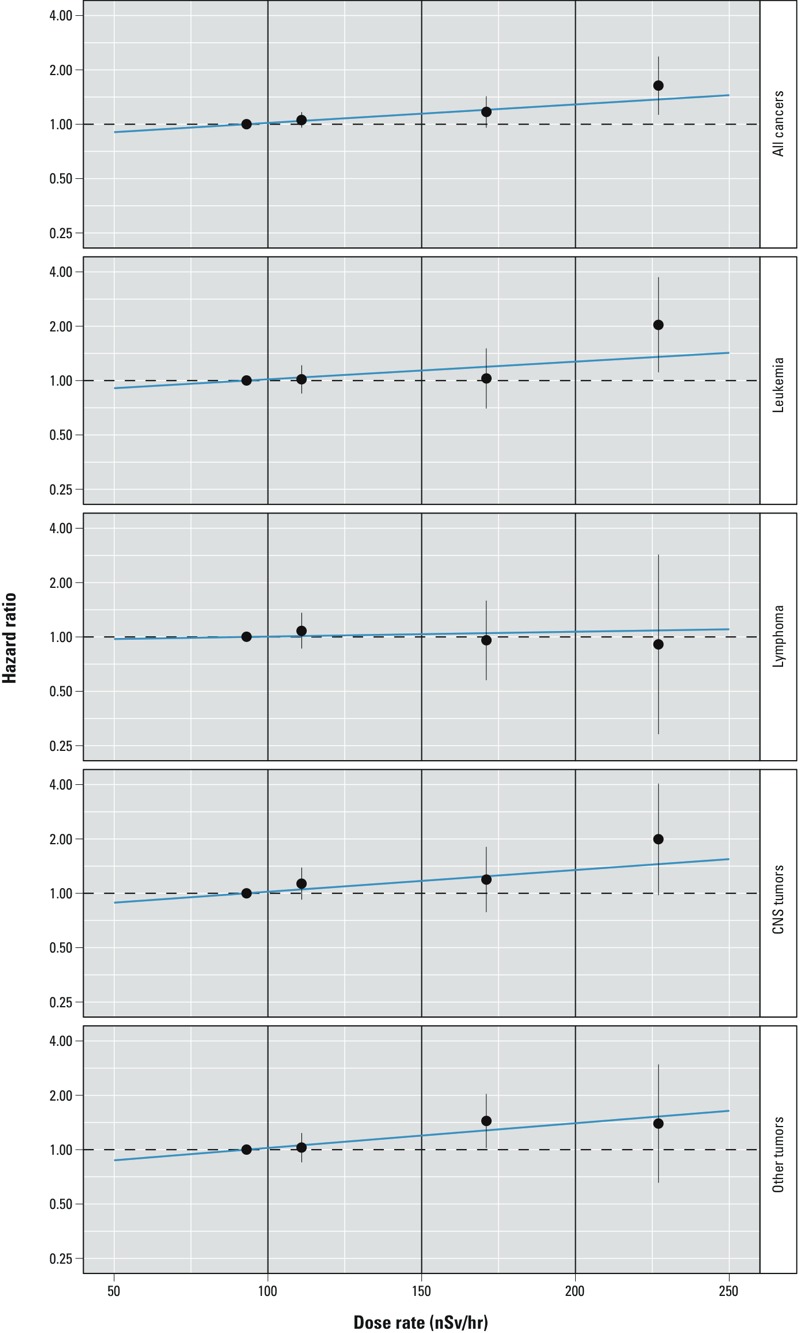
Hazard ratios for cancer by dose rate of external ionizing radiation among children < 16 years of age in the Swiss National Cohort. Results from Cox proportional hazards models adjusting for sex and birth year using a categorized exposure [points and bars (95% CIs) placed along the *x*-axis at mean dose rates within categories; categories delineated by vertical lines] and a linear exposure term (blue line). Dose rates < 100 nSv/hr are the reference category.

*Association between childhood cancer and dose rate*. Tables 2 and 3 and Figure 2 show results of analyses using dose rate as exposure. We found a markedly increased risk among children exposed to a dose rate ≥ 200 nSv/hr compared with those exposed to < 100 nSv/hr for any cancer [hazard ratio (HR) = 1.64; 95% confidence interval (CI): 1.13, 2.37], leukemia (HR = 2.04; 95% CI: 1.11, 3.74), ALL (HR = 2.12; 95% CI: 1.09, 4.16), and CNS tumors (HR = 1.99; 95% CI: 0.98, 4.05) (Table 2). For intermediate exposure levels, HRs tended to be close to 1. Adjusting for potential confounders did not materially alter results (see Supplemental Material, Figure S2). In trend analyses using a linear exposure term, HRs per increase of 100 nSv/hr in dose rate were between 1.2 and 1.4 for all diagnostic groups except lymphoma, where it was close to 1. The lower confidence limit exceeded 1 only for all cancers (HR = 1.27; 95% CI: 1.06, 1.52 per 100 nSv/hr) (Table 3, Figure 2). When we restricted analyses to children with stable residence before entry into the cohort (66.5% of the entire cohort), results remained similar with somewhat larger effect estimates (Table 3; see also Supplemental Material, Table S2).

**Table 2:** Association between childhood cancer and dose rate of external background radiation in the Swiss National Cohort.

Outcome	Dose rate (nSv/hr)	Cases (*n*)	IR^*a*^	HR (95% CI)^*b*^
All cancers	< 100	659	10.56	1.00 (reference)
	100 to < 150	982	11.16	1.06 (0.96, 1.17)
	150 to < 200	112	12.32	1.17 (0.96, 1.43)
	≥ 200	29	17.22	1.64 (1.13, 2.37)
Leukemia	< 100	201	3.22	1.00 (reference)
	100 to < 150	288	3.27	1.02 (0.85, 1.22)
	150 to < 200	30	3.30	1.03 (0.70, 1.51)
	≥ 200	11	6.53	2.04 (1.11, 3.74)
ALL	< 100	158	2.53	1.00 (reference)
	100 to < 150	225	2.56	1.01 (0.82, 1.24)
	150 to < 200	24	2.64	1.05 (0.68, 1.61)
	≥ 200	9	5.34	2.12 (1.09, 4.16)
Lymphoma	< 100	122	1.96	1.00 (reference)
	100 to < 150	186	2.11	1.08 (0.86, 1.36)
	150 to < 200	17	1.87	0.96 (0.58, 1.59)
	≥ 200	3	1.78	0.91 (0.29, 2.86)
CNS tumors	< 100	150	2.40	1.00 (reference)
	100 to < 150	239	2.72	1.13 (0.92, 1.39)
	150 to < 200	26	2.86	1.19 (0.79, 1.81)
	≥ 200	8	4.75	1.99 (0.98, 4.05)
Other malignant tumors	< 100	186	2.98	1.00 (reference)
	100 to < 150	269	3.06	1.03 (0.85, 1.24)
	150 to < 200	39	4.29	1.44 (1.02, 2.04)
	≥ 200	7	4.16	1.39 (0.66, 2.97)
Abbreviations: ALL, acute lymphoblastic leukemia; CNS, central nervous system; HR, hazard ratio; IR, incidence rate.^***a***^Per 100,000 person-years at risk. ^***b***^From Cox proportional hazards models adjusting for sex and birth year.				

**Table 3:** Hazard ratios for childhood cancer per 100 nSv/hr dose rate of external radiation in the Swiss National Cohort.

Outcome	Entire cohort		Subcohort with stable place of residence before entry^*a*^	
	HR (95% CI)^*b*^	*p*-Value	HR (95% CI)^*b*^	*p*-Value
All cancers	1.27 (1.06, 1.52)	0.011	1.33 (1.08, 1.62)	0.006
Leukemia	1.25 (0.90, 1.75)	0.186	1.31 (0.90, 1.92)	0.159
ALL	1.23 (0.84, 1.81)	0.278	1.31 (0.86, 2.01)	0.205
Lymphoma	1.06 (0.68, 1.67)	0.788	1.16 (0.70, 1.92)	0.558
CNS tumors	1.32 (0.91, 1.91)	0.139	1.42 (0.96, 2.12)	0.081
Other malignant tumors	1.37 (0.98, 1.91)	0.064	1.36 (0.93, 1.98)	0.110
Abbreviations: ALL, acute lymphoblastic leukemia; CNS, central nervous system; HR, hazard ratio.^***a***^Children with same residence 5 years before entry into the cohort or, if this information was lacking, lived in the same municipality at birth. ^***b***^From Cox proportional hazards models using a linear exposure term adjusting for sex and birth year.				

*Trend analyses using cumulative dose*. Calculated cumulative dose had a mean of 9.06 mSv (median, 9.12 mSv; range, 0.03–49.4 mSv; interquartile range, 5.55–12.1 mSv) and, as expected, correlated strongly with age (see Supplemental Material, Table S3). Trend analyses using cumulative dose showed a similar pattern as for dose rate with stronger evidence of a trend for all cancers (HR = 1.028; 95% CI: 1.008, 1.048 per mSv cumulative dose), leukemia (HR = 1.036; 95% CI: 0.997, 1.077), and CNS tumors (HR = 1.042; 95% CI: 1.002, 1.084) (Table 4). Restricting to the subcohort with stable place of residence before census again resulted in larger effect estimates, particularly for CNS tumors (HR = 1.060; 95% CI: 1.015, 1.106 per mSv) (Table 4).

**Table 4:** Hazard ratios for childhood cancer per mSv cumulative dose of external radiation in the Swiss National Cohort.

Outcome	Entire cohort		Subcohort with stable placeof residence before entry^*a*^	
	HR (95% CI)^*b*^	*p*-Value	HR (95% CI)^*b*^	*p*-Value
All cancers	1.028 (1.008, 1.048)	0.006	1.040 (1.017, 1.064)	< 0.001
Leukemia	1.036 (0.997, 1.077)	0.075	1.046 (0.999, 1.096)	0.054
ALL	1.037 (0.990, 1.086)	0.124	1.049 (0.994, 1.107)	0.084
Lymphoma	1.007 (0.964, 1.052)	0.746	1.022 (0.973, 1.073)	0.386
CNS tumors	1.042 (1.002, 1.084)	0.041	1.060 (1.015, 1.106)	0.008
Other malignant tumors	1.025 (0.989, 1.063)	0.177	1.034 (0.991, 1.078)	0.121
Abbreviations: ALL, acute lymphoblastic leukemia; CNS, central nervous system; HR, hazard ratio. ^***a***^Children with same residence 5 years before entry into the cohort or, if this information was lacking, lived in the same municipality at birth. ^***b***^From conditional logistic regression in nested case–control sample (equivalent to Cox proportional hazards regression) adjusting for sex and birth year.				

## Discussion

This nationwide census-based cohort study in Switzerland found evidence of an increased risk of cancer among children exposed to external dose rates of background ionizing radiation of ≥ 200 nSv/hr compared with those exposed to < 100 nSv/hr. Trend analyses showed an increasing risk with cumulative dose received since birth for all cancers taken together, and for leukemia and CNS tumors.

Although the effects of acute exposure to moderate or high doses (> 100 mSv) of ionizing radiation on the risk of childhood cancer have been clearly demonstrated, the evidence for protracted exposure to low-dose radiation is still scarce (Wakeford 2013). Studies from high-risk groups, including atomic bomb survivors and groups exposed to therapeutic radiation, report relative risks in the order of about 5–8 and 2–5 per Sv for leukemia and CNS tumors, respectively, among subjects exposed at < 20 years of age (UNSCEAR 2013). For leukemia, estimated relative risks exceed 50 per Sv shortly after exposure among those exposed in early life (UNSCEAR 2013; Wakeford 2013). Extrapolating from models calibrated to risks observed in atomic bomb survivors, the excess fraction of childhood leukemia cases due to natural background radiation has been estimated to be up to about 20% in France (Laurent et al. 2013) and the United Kingdom (Little et al. 2009). However, there are great uncertainties attached to such estimates.

A recent register-based case–control study from the United Kingdom including 27,447 cases (of whom 9,058 had leukemia and 6,585 had CNS tumors) and 36,793 matched controls found a relative risk of 1.03 (95% CI: 1.00, 1.07) for all childhood cancer, 1.09 (95% CI: 1.02, 1.17) for leukemia, 1.10 (95% CI: 1.02, 1.19) for ALL, 1.01 (95% CI: 0.93, 1.09) for lymphoma, and 1.02 (95% CI: 0.96, 1.08) for CNS tumors for each milligray increase in cumulative indoor gamma-ray exposure since birth (Kendall et al. 2013). Given the rarity of childhood cancer, the hazard ratios per millisievert increase in cumulative dose of outdoor radiation found in our study (Table 4) can be interpreted as risk ratios (Symons and Moore 2002). Assuming a 20% reduction of doses due to the shielding effect of buildings (UNSCEAR 2000), an estimated difference of 1 mSv cumulative dose in our data relates to a difference indoors of 0.8 mSv, and the results in the first column of Table 4 translate to relative risks of 1.03 (95% CI: 1.01, 1.06) for all childhood cancers, 1.05 (95% CI: 1.00, 1.10) for leukemia, 1.05 (95% CI: 0.99, 1.11) for ALL, 1.01 (95% CI: 0.96, 1.07) for lymphoma, and 1.05 (95% CI: 1.00, 1.11) for CNS tumors. Compared to the UK study, our point estimates are smaller for leukemia and larger for CNS tumors. However, the wide overlap of confidence intervals for corresponding outcomes demonstrates good agreement between the studies. The effect estimates observed in the population with stable residence before entry into the cohort (Table 4) suggest that failure to account for residential mobility results in a downward bias and that relative risks in both studies were underestimated. Somewhat surprisingly, our confidence intervals are narrower despite the much smaller number of cases included in the study. This could have several reasons: For a given number of cases, a cohort study such as ours has larger statistical power than a case–control study with only a single control per case (Little et al. 2010); both the case–control pairs in the UK study and the risk sets in our study were age-matched; and, conditional on age, variability of cumulative exposure was considerably larger in our study [compare Supplemental Material, Table S3, with Table S7 in Kendall et al. (2013)]; furthermore, the UK study assessed exposures with a lower spatial resolution, and, consequently, almost half of the cases shared the same exposure level as their controls.

Few other studies have used individual data to investigate a potential link between childhood cancer and radiation from natural gamma or cosmic rays (Axelson et al. 2002; UK Childhood Cancer Study Investigators 2002b) (see Supplemental Material, Table S4). The UK Childhood Cancer Study—a case–control study that used gamma dose rates (including the cosmic component of penetrating radiation) measured in children’s homes at the time of diagnosis—found no evidence of an association for leukemia. As in our analysis, effect estimates for CNS tumors were elevated in the highest exposure categories, but confidence intervals were wide and included one. A case–control study from Sweden reported an odds ratio of 1.4 (95% CI: 1.0, 2.0) for ALL among subjects < 20 years of age, comparing those living in buildings made of alum shale concrete with those living in other houses (Axelson et al. 2002). Overall, studies using individual data suggest an increasing risk for ALL with cumulative dose of natural gamma radiation (see Supplemental Material, Table S4). In contrast to this, an investigation in high background radiation areas in China and India found no indication of increased risks of childhood leukemia (Akiba et al. 2002).

We did not adjust for domestic radon exposure because this information was available only for the 2000 census, and our recent study on domestic radon and childhood cancer found no evidence of an association in the SNC (Hauri et al. 2013). The UK record-based case–control study by Kendall et al. and the UK Childhood Cancer Study also found little indication of an increased risk of childhood cancers due to radon (Kendall et al. 2013; UK Childhood Cancer Study Investigators 2002a). However, a Danish record-based case–control study and most ecologic studies reported positive associations between childhood leukemia and domestic radon (Raaschou-Nielsen 2008; Raaschou-Nielsen et al. 2008; Tong et al. 2012).

Exposure assessment in our study was based on a geographic model rather than on actual measurements at children’s homes. Although the model was based on a dense net of measurements covering the entire country, methods of interpolation and calibration, measurement error, and the neglect of exposure variability due to natural factors such as snow cover or sun activity are likely to have caused some exposure misclassification. Calculated doses were based on outdoor dose rates, although children spend most of their time indoors. Unfortunately we did not have address histories for the entire population and could therefore not fully account for residential mobility in our calculation of cumulative dose. However, for some children (21%) residential locations were known at two time points. Outcome assessment was based on probabilistic record linkage between the SCCR and SNC and is likely to have resulted in some misclassification of the outcomes. Based on linkage results, we judge that at least 93% of the linked SCCR–SNC pairs represent true matches. These either had residential locations matching to within 50 m combined with perfect matches on date of birth, sex, and municipality of residence at birth or had more convincing similarities. Assuming that half of the remaining pairs are false matches, < 4% of those classified as having cancer were false positives. Conversely, > 400 potentially incident cases were not linked to the SNC, likely resulting in false negatives. But we found no indication that these differed from linked cases in radiation exposure, suggesting that the risk of bias due to linkage errors was small.

Major strengths of our study are its cohort design and the use of nationwide routine data. The cohort design maximizes statistical power for a given number of cases and accounts for lost to follow-up by migration or death. Assessment of both exposures and outcomes were based on routine data of nationwide coverage essentially eliminating the risk of selection bias. Our study was able to include a wide range of potential confounding factors. Furthermore, our study was based on a relatively wide range of exposure levels.

It is plausible that the observed associations between background radiation and childhood cancer reflect a causal relationship: Ionizing radiation is known to cause childhood cancer at high doses and dose rates. Associations were stronger for outcomes previously linked to radiation, such as leukemia and CNS tumors, although no evidence of an association was found for lymphoma where such links have been less demonstrable (UNSCEAR 2006). Our findings were little affected by adjustments for a number of potentially confounding factors. We found evidence of a dose response, and this evidence was strongest in a subcohort of children with stable residence before entry into the cohort, that is, with less exposure misclassification due to residential mobility. We cannot, however, exclude biases due to inaccurate exposure measurement.

In conclusion, our study suggests that background radiation may contribute to the risk of cancer in children. Results suggest that risks for leukemia and CNS tumors are similarly affected. Future research in this field could greatly benefit from improved exposure assessment.

## Supplemental Material

(495 KB) PDF
