# Supplementary material for: Background Ionizing Radiation and the Risk of Childhood Cancer: A Census-Based Nationwide Cohort Study
Source: Environ Health Perspect. 2015 Feb 23;123(6):622–8. doi: 10.1289/ehp.1408548 (PMC4455589; doi:10.1289/ehp.1408548)
Supplement: (495 KB) PDF [file ehp.1408548.s001.508.pdf]

Environmental Health Perspectives doi:10.1289/ehp.1408548

Supplemental Material

Background Ionizing Radiation and the Risk of Childhood Cancer: A Census-Based Nationwide Cohort Study

Ben D. Spycher, Judith E. Lupatsch, Marcel Zwahlen, Martin Röösli, Felix Niggli, Michael A. Grotzer, Johannes Rischewski, Matthias Egger, and Claudia E. Kuehni for the Swiss Pediatric Oncology Group and the Swiss National Cohort Study Group

Table S1. Characteristics of cancer cases included in analyses.

Characteristics	Included in time to event analysis N (%)	Total eligible N (%)
**All cases**	1,782 (100.0)	3,502 (100.0)
**ICCC3 main diagnostic group**		
Leukemias, myeloproliferative diseases, and myelodysplastic diseases	530 (29.7)	1,092 (31.2)
Lymphomas and reticuloendothelial neoplasms	328 (18.4)	474 (13.5)
CNS and miscellaneous intracranial and intraspinal neoplasms	423 (23.7)	775 (22.1)
Neuroblastoma and other peripheral nervous cell tumors	34 (1.9)	220 (6.3)
Retinoblastoma	21 (1.2)	95 (2.7)
Renal tumors	55 (3.1)	175 (5.0)
Hepatic tumors	14 (0.8)	40 (1.1)
Malignant bone tumors	127 (7.1)	176 (5.0)
Soft tissue and other extraosseous sarcomas	123 (6.9)	237 (6.8)
Germ cell tumors, trophoblastic tumors, and neoplasms of gonads	48 (2.7)	98 (2.8)
Other malignant epithelial neoplasms and malignant melanomas or unspecified malignant neoplasms	79 (4.4)	111 (3.2)
**Sex**		
Male	993 (55.7)	1945 (55.5)
Female	789 (44.3)	1557 (44.5)
**Median age in years (IQR)**	10.0 (6.0, 13.2)	6.2 (2.7, 11.8)

Abbreviations: ICCC3 International Classification of Childhood Cancers – 3rd Edition (Steliarova-Foucher et al. 2005), IQR interquartile range, CNS central nervous system.

Table S2. Association between childhood cancer and dose rate of external ionizing radiation among children with stable place of residence[a] before entry into Swiss National Cohort.

Dose rate	Cases	IR[b]	HR (95% CI)[c]
**All cancers**			
<100 nSv/h	470	11.24	1.00[d]
100 - <150 nSv/h	726	12.22	1.09 (0.97, 1.22)
150 - <200 nSv/h	89	13.94	1.24 (0.99, 1.56)
≥200 nSv/h	26	20.33	1.82 (1.23, 2.70)
**Leukemia**			
<100 nSv/h	141	3.37	1.00[d]
100 - <150 nSv/h	210	3.54	1.05 (0.84, 1.29)
150 - <200 nSv/h	22	3.45	1.03 (0.66, 1.61)
≥200 nSv/h	9	7.04	2.13 (1.09, 4.18)
**ALL**			
<100 nSv/h	112	2.68	1.00[d]
100 - <150 nSv/h	169	2.85	1.06 (0.83, 1.34)
150 - <200 nSv/h	19	2.98	1.12 (0.69, 1.82)
≥200 nSv/h	7	5.47	2.09 (0.98, 4.50)
**Lymphoma**			
<100 nSv/h	84	2.01	1.00[d]
100 - <150 nSv/h	131	2.21	1.10 (0.84, 1.45)
150 - <200 nSv/h	14	2.19	1.09 (0.62, 1.92)
≥200 nSv/h	3	2.35	1.15 (0.36, 3.65)
**CNS tumors**			
<100 nSv/h	111	2.65	1.00[d]
100 - <150 nSv/h	185	3.11	1.17 (0.93, 1.49)
150 - <200 nSv/h	21	3.29	1.24 (0.78, 1.97)
≥200 nSv/h	8	6.26	2.37 (1.16, 4.86)
**Other malignant tumors**			
<100 nSv/h	134	3.20	1.00[d]
100 - <150 nSv/h	200	3.37	1.05 (0.84, 1.31)
150 - <200 nSv/h	32	5.01	1.57 (1.07, 2.31)
≥200 nSv/h	6	4.69	1.47 (0.65, 3.33)

Abbreviations: ALL acute lymphoblastic leukemia, CNS central nervous system, IR incidence rate, HR hazard ratio, CI confidence interval.

[a]Children with same residence 5 years before census or, if this information was lacking, lived in the same municipality at birth. [b]Incidence rates are per 100,000 person years at risk. [c]From Cox proportional hazard models adjusting for sex and birth year. [d]Reference category.

Table S3. Numbers of observations according to category of cumulative dose of external ionizing radiation and age in a nested cases-control sample of children from the Swiss National Cohort.

Age (years)	[0-2) mSv	[2-4) mSv	[4-6) mSv	[6-8) mSv	[8-10) mSv	[10-12) mSv	[12-14) mSv	[14-16) mSv	[16-18) mSv	[18-20) mSv	[20-22) mSv	[22-24) mSv	[24-26) mSv	[26-28) mSv	[28-30) mSv	≥30 mSv	Total
<1	1411	0	0	0	0	0	0	0	0	0	0	0	0	0	0	0	1411
1	4461	377	3	0	0	0	0	0	0	0	0	0	0	0	0	0	4841
2	1937	6004	223	8	0	0	0	0	0	0	0	0	0	0	0	0	8172
3	10	8749	959	151	13	0	0	0	0	0	0	0	0	0	0	0	9882
4	0	3961	4455	458	93	12	1	0	0	0	0	0	0	0	0	0	8980
5	0	370	9147	981	409	74	12	3	0	0	0	0	0	0	0	0	10996
6	0	19	6220	3653	537	276	55	15	1	1	0	0	0	0	0	0	10777
7	0	2	1758	8208	1019	419	195	46	20	7	0	0	0	0	0	0	11674
8	0	1	130	7805	3096	563	348	164	38	17	8	0	1	0	0	0	12171
9	0	0	19	2885	5755	792	371	271	104	44	15	9	4	1	1	0	10271
10	0	0	8	609	8326	2943	630	367	304	100	43	24	15	1	0	3	13373
11	0	0	0	80	4988	6819	1097	496	360	235	104	48	16	12	3	6	14264
12	0	0	0	25	1988	8053	2724	635	351	324	225	75	41	17	8	9	14475
13	0	0	0	18	360	7562	6478	1189	617	366	330	212	69	32	20	33	17286
14	0	0	0	0	89	3407	7709	2799	728	423	314	286	162	58	38	38	16051
15	0	0	0	2	31	943	6628	4819	1048	518	306	272	222	115	55	82	15041
**Total**	7819	19483	22922	24883	26704	31863	26248	10804	3571	2035	1345	926	530	236	125	171	179665

Table S4. Summary of studies on childhood cancer and background gamma radiation using individual level data.

Study	Design	Country	Age (yrs)	Period diagnosed	No. Cases	Type of radiation	Exposure assessment	Exposure timing	ALL<br>Dose rate: RR (95% CI)	ALL<br>Cumulative dose: RR (95% CI)
(Axelson et al. 2002)	Case-control	Sweden	<20	1980-1989	312[a]	Gamma radiation from building facades	Residence in an alum shale concrete house[b]	Based on full residential history	Ever in alum shale house vs. never[b]<br>1.4 (CI 1.0, 2.0)	Never: referent<br>Low: 1.3 (0.84, 2.1)<br>High: 1.5 (0.98, 2.3)
(UKCCS Investigators 2002)	Case-control	Great Britain	<15	1991-1996	2,165	Indoor terrestrial and cosmic[c]	Measure-ments in children's homes	At diagnosis	<5650.3 µGy/y: Ref.<br>650.3–5798.5 µGy/y: 1.00 (0.72, 1.40)<br>798.5–5916.2 µGy/y: 1.20 (0.85, 1.68)<br>916.2–41045.3 µGy/y: 0.96 (0.67, 1.37)<br>≥1045.3 µGy/y: 0.93 (0.65, 1.33)	
(Kendall et al. 2013)	Record-based case-control	Great Britain	<15	1980-2006	27,447	Indoor terrestrial and cosmic[c]	Mean dose rates within county district	At birth		1.10 (1.02, 1.19) per mGy
Present study	Census-based cohort	Switzerland	<16	1990-2008	1,782	Outdoor terrestrial and cosmic[c]	Geographic model	At census 1990, updated at census 2000	1.23 (0.84, 1.81) per 100 nSv/h	1.04 (1.00, 1.09) per mSv

Abbreviations: ALL acute lymphoblastic leukemia, CI confidence interval, RR relative risk, UKCCS UK Childhood Cancer Study.

[a]Includes only ALL cases. [b]Gamma-radiation dose rates are assumed to be 300 nSv/h inside alum shale concrete houses and 100 nSv/h inside other houses.

[c]Terrestrial gamma radiation and the directly ionizing component of cosmic radiation.

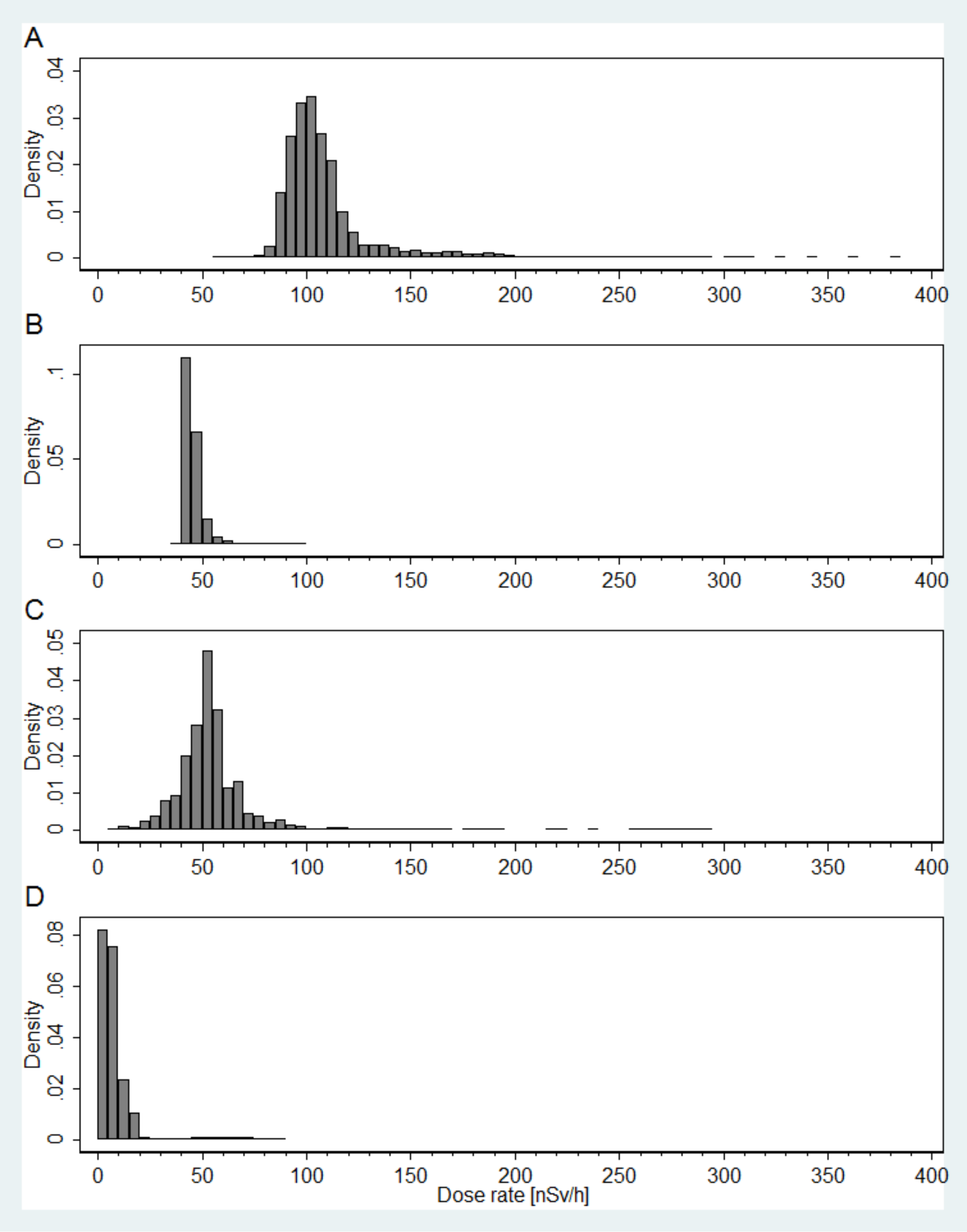

Figure S1. Distribution of dose rate from all sources (Total) (A), cosmic rays (B), natural terrestrial (C), and artificial terrestrial radiation (D) among children of the Swiss National Cohort.

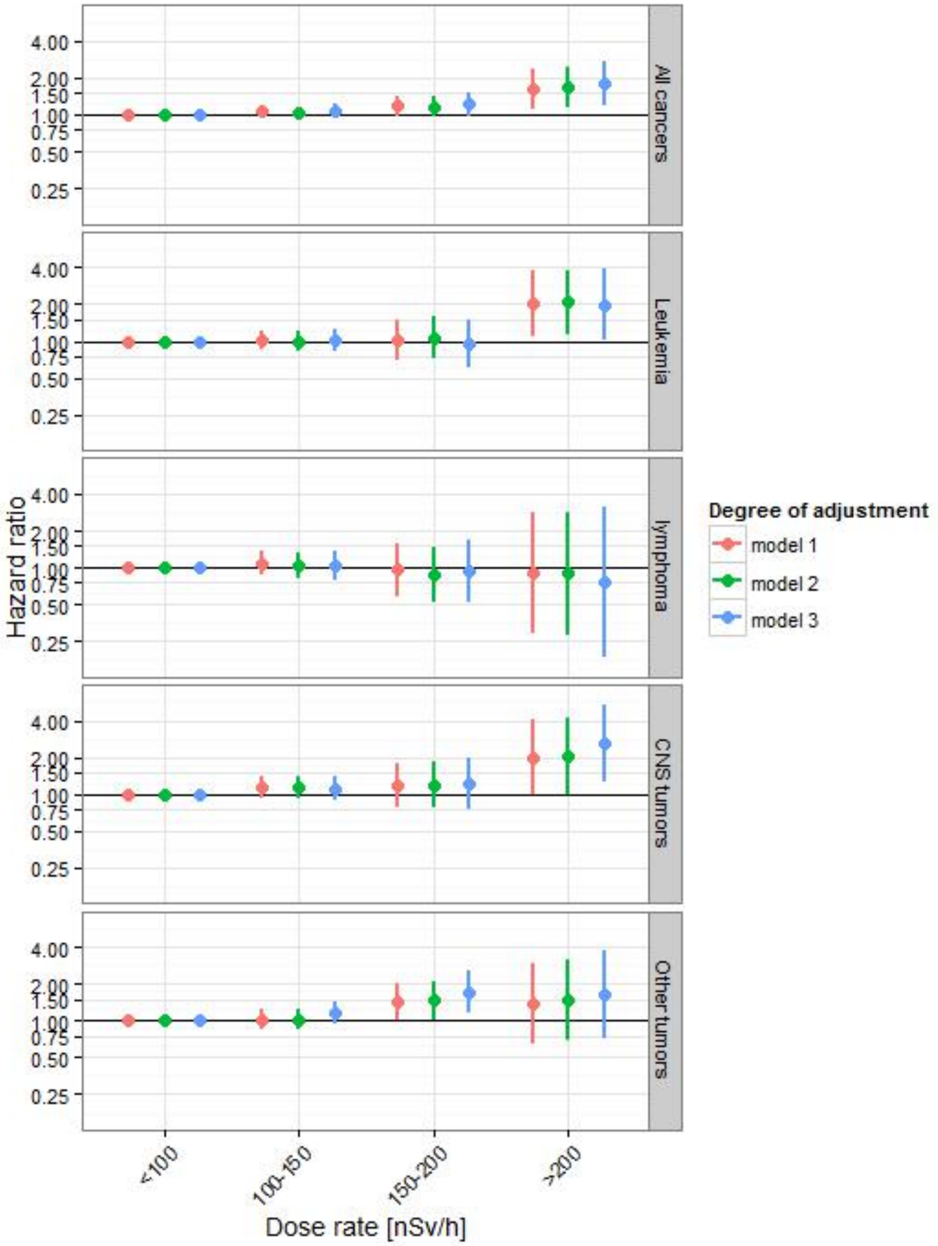

Figure S2. Effects on hazard ratios of adjusting for potential confounders. Results from Cox proportional hazards regression. Model 1: adjusting for sex and birth year. Model 2: additionally adjusting for distance to nearest highway, predicted field strength from broad cast transmitters based on a geographic model, distance to nearest high voltage power line, degree of urbanization of municipality, neighborhood socio-economic status (Panczak et al. 2012), education of household reference person and crowding. Model 3: adjusting for all the previous and for birth weight and birth order.

References

Axelson O, Fredrikson M, Akerblom G, Hardell L. 2002. Leukemia in childhood and adolescence and exposure to ionizing radiation in homes built from uranium-containing alum shale concrete. Epidemiology 13:146-150.

Kendall GM, Little MP, Wakeford R, Bunch KJ, Miles JC, Vincent TJ, et al. 2013. A record-based case-control study of natural background radiation and the incidence of childhood leukaemia and other cancers in Great Britain during 1980-2006. Leukemia 27:3-9.

Panczak R, Galobardes B, Voorpostel M, Spoerri A, Zwahlen M, Egger M, et al. 2012. A Swiss neighbourhood index of socioeconomic position: development and association with mortality. J Epidemiol Community Health 66:1129-1136.

Steliarova-Foucher E, Stiller C, Lacour B, Kaatsch P. 2005. International Classification of Childhood Cancer, third edition. Cancer 103:1457-1467.

UKCCS Investigators. 2002. The United Kingdom Childhood Cancer Study (UKCCS) of exposure to domestic sources of ionising radiation: 2: gamma radiation. Br J Cancer 86:1727-1731.
